# Application of Chia and Flaxseed Meal as an Ingredient of Fermented Vegetable-Based Spreads to Design Their Nutritional Composition and Sensory Quality

**DOI:** 10.3390/foods14030438

**Published:** 2025-01-29

**Authors:** Katarzyna Waszkowiak, Krystyna Szymandera-Buszka, Marcin Kidoń, Joanna Kobus-Cisowska, Anna Brzozowska, Angelika Kowiel, Maciej Jarzębski, Elżbieta Radziejewska-Kubzdela

**Affiliations:** 1 Department of Gastronomy Science and Functional Foods, Faculty of Food Science and Nutrition, Poznań University of Life Sciences, Wojska Polskiego 31, 60-624 Poznań, Poland; krystyna.szymandera_buszka@up.poznan.pl (K.S.-B.); joanna.kobus-cisowska@up.poznan.pl (J.K.-C.); anna.brzozowska@up.poznan.pl (A.B.); 2 Department of Food Technology of Plant Origin, Faculty of Food Science and Nutrition, Poznań University of Life Sciences, Wojska Polskiego 31, 60-624 Poznań, Poland; marcin.kidon@up.poznan.pl (M.K.); agelikaa90@gmail.com (A.K.); 3 Department of Physics and Biophysics, Faculty of Food Science and Nutrition, Poznań University of Life Sciences, Wojska Polskiego 38/42, 60-637 Poznań, Poland; maciej.jarzebski@up.poznan.pl

**Keywords:** lactic fermentation, chia seed, flax seed, cucumber, zucchini, chemical composition, sensory quality, phenolic compounds, antioxidant activity, instrumental analysis

## Abstract

Fermented vegetable spreads could offer an opportunity to diversify the range of plant-based foods. The challenge in developing the spreads is to achieve high quality, including stable consistency, consumer desirability and high nutritional value. The aim was to evaluate the application of chia and flaxseed meal for fermented zucchini-cucumber spread production. The effect on the chemical composition, phenolic compound content, antioxidant activity, and sensory quality of the vegetable spread was evaluated. Its color, viscosity, and microstructure were also analyzed using instrumental methods. The meal addition varied from 4.0 to 14.0%. The spread with meal addition had higher fat, protein, ash, and dietary fiber content than the control. Total free phenolic compound content and antioxidant activity also increased, and chia seed meal impacted the parameters more. On the contrary, flaxseed meal improved more the product’s consumer desirability than chia. Both were effective gelling agents that increased viscosity and enhanced product spreadability, and only flaxseed meal showed a masking ability. Its addition reduced the perception and intensity of the bitter, tart, and sour taste. The spread formula consisting of fermented zucchini and cucumber with 9 to 11.5% flaxseed meal addition was the most recommended to achieve the product with high consumer desirability.

## 1. Introduction

Consumption of fermented vegetables with unique sensory attributes can diversify consumers’ daily diets and improve human health [1]. Probiotic bacteria and compounds cumulated or degraded during fermentation are crucial for the health benefits of fermented vegetables. Among the others, the metabolic activities of fermenting microbiota can realize plant compounds or transform them into biologically active substances. Fermented vegetables are a source of valuable compounds, including short-chain fatty acids, γ-aminobutyric acid, minerals, B vitamins and phenolic compounds with antioxidant activity [2]. Therefore, fermented vegetable-based products can be a valuable group of food.

Fermented foods, including vegetables and vegetable-based products, are an important segment of the food market. The food industry is still searching for new ways to use fermented vegetables in food production and design new products. A spread based on fermented vegetables can be a new, healthy and attractive-to-customer alternative to other vegan and non-vegan spreads, broadening the range of vegan products on the market.

One of the challenges in developing vegetable-based spreads is to achieve their desired and stable consistency. The solution is to use various plant-originated hydrocolloids. Among them, a mucilaginous constituent of chia seed (*Salvia hispanica* L.) or flax seed (*Linum usitatissimum* L.) coat may show potential in such products development. Mucilages consist of soluble substances or substances that swell in the presence of water [3] which exhibit a high water-binding capacity and can form a viscous gel even at low temperatures [4]. From a nutritional point of view, carbohydrates forming plant mucilage are classified as soluble dietary fiber. Flaxseed mucilage is mostly compost of a complex heteropolysaccharides of high molecular weight, comprising two fractions: a neutral arabinoxylan and rhamnogalacturonan [5,6]. It also contains about 9% protein. Flaxseed mucilage has excellent functional properties such as water-holding capacity, oil-holding capacity, emulsifying capacity, foaming capacity, and stability [7]. The largest component of chia mucilage are carbohydrates mainly consisting of arabinose and xylose (85%) [8]. Chia seed mucilage has high water solubility and forms viscous gels at low concentrations, high emulsifying capacity and water retention [9,10]. Moreover, chia and flaxseed proteins can also be crucial hydrocolloids for achieving the desired spread consistency due to their valuable functional properties [11,12].

Fermented vegetables characterize unique flavors due to production and accumulation of volatile and non-volatile aroma or aroma active compounds during the process, including those related to bitter, sweet, sour, and salty tastes [13]. Certain groups of consumers do not prefer a fermented vegetable flavor [14]. Thus, designing new fermented vegetable-based food may also require modification or even masking of the characteristic taste to be more desirable for consumers. Plant hydrocolloids, such as those present in chia and flax seeds, have the potential to be flavor maskers [15].

Except mucilage and proteins, flax and chia seeds are also a source of other macro- and micronutrients and phenolic compounds. Therefore, their introduction into vegetable-based spreads may also enhance nutritional quality and antioxidant capacity of such products. Both seeds are reach in dietary fiber and oil with high n-3 unsaturated fatty acid share [16,17]. Chia seeds are rich in rosmarinic acid, daidzein, quercetin, and flax seeds in lignan secoisolariciresinol and phenolic acids. [18,19]. These compounds exhibit high antioxidant capacity, crucial for various health benefits related to reducing the risk and preventing certain diseases. Phenolic acids and flavonoids identified in the seeds show anti-inflammatory, antimicrobial, or anticancer properties [20]. Flax seeds lignan secoisolariciresinol is reported as a key compound in preventing hormone-dependent cancers [21], diabetes, and cardiovascular diseases [22].

In the study, the application of chia and flaxseed meals as an ingredient of fermented vegetable (cucumber and zucchini) based spreads was studied for the first time. The hypothesis was tested; the meal additions improved the spreads nutritional composition and sensory quality. The variable meal addition (between 4% and 14%) to the vegetable-based spreads was applied. Moreover, the relationships between changes in the vegetable spread physicochemical properties (i.e., color and viscosity) analyzed with instrumental methods and consumer desirability were evaluated to examine the possibility of using the instrumental tests to plan the direction of vegetable spread development. The spread microstructure with microscopic analysis was also evaluated.

## 2. Materials and Methods

### 2.1. Fermented Vegetables and Reagents

Cucumber (*Cucumis sativus* L.) and zucchini (*Cucurbita pepo* L. morphotype Zucchini) were provided by local suppliers in the 2023 season. Cucumbers were fermented on the farm (Gospodarstwo Rolne Marek Biesiada, Jastrzębniki, Poland). Fresh cucumbers were washed in the brush washer, put in plastic barrels of volume 200 L, about 120 kg each, seasoned (dried garlic, dill, oak leaf), and poured in about 80 L of salt brine (7% of sodium chloride in water). Spontaneous fermentation took place at 18 °C for the first 14 days, then the temperature was reduced to 14 °C for the next 4 weeks and finally fermented cucumbers were stored at 6 °C. Fermented cucumbers were separated from brine and sorted. Curved, broken, overgrown, or undergrown cucumbers unsuitable for culinary consumption were homogenized and used for vegetable spread production.

Zucchini fermentation was conducted in the plant of Runoland Company (Runoland, Góra, Poland). The raw materials were washed using an industrial vegetable brush washer. The washer was equipped with a 2 × 4 kW ultrasonic generator with a frequency of 25 kHz. During zucchini washing, ultrasound waves were applied for 5 min. After washing, the zucchinis were cut into slices about 1 cm thick and placed in buckets lined with plastic bags. Then, a brine consisting of 3% sodium chloride in water was added. The ratio of vegetables to brine was approximately 1:1. Lactic acid bacteria (LAB) probiotic strains *Lactiplantibacillus plantarum* 299 v was added to improve the fermentation process. The commercially available freeze-dried microorganisms (Sanprobi, Szczecin, Poland) were dissolved in the brine and added to buckets. The concentration of microorganisms was 5 log CFU g^−1^ of vegetables. The buckets with zucchini were sealed and placed at 35 °C for 7 days of fermentation. After this time, the zucchini slices were separated by a sieve from the brine and homogenized to a puree and in this form used for vegetable spread production.

Flaxseed (*Linum usitatissimum* L.) meal and chia (*Salvia hispanica* L.) seeds were purchased from a local producer (Sante, Warszawa, Poland). The flaxseed meal was a ground flaxseed cake of golden seed cultivars (a by-product of the cold pressing of oil). Their declared chemical compositions were as follows (per 100 g): 32 g and 23 g of protein, 12 g and 34 g of fat, 32 g and 34 g of dietary fiber for flaxseed meal and chia seeds, respectively. The chia seeds were ground with a WZ-1 laboratory grinder (Sadkiewicz Instruments, Bydgoszcz, Poland) to prepare chia seed meal. The granulation of the meals was below 350 µm.

All reagents and solvents (analytical (ACS) or HPLC grade) were purchased from POCH (Gliwice, Poland) or Merck (Darmstadt, Germany).

### 2.2. Fermented Vegetable-Based Spread Preparation

In the study, we intended to develop a product that could help manage fermented cucumbers unsuitable for culinary use, i.e., crooked, broken, overgrown, or undergrown. Accordingly, it was assumed that the designed zucchini-cucumber spread would be based on fermented cucumber, and the control recipe would contain 50% of this raw material. The spread samples were prepared according to the formulations presented in Table 1. Fermented cucumber and fermented zucchini purees were mixed in the ratio 1:1 using Thermomix^®^TM5 (Vorwerk, Wroclaw, Poland). Flaxseed or chia seed meal was then added, and the mixture was mixed for a further 5 min until a homogeneous consistency was obtained. The variance factor that changed the composition and quality of the vegetable spreads was the addition of the meals, which constituted 4.0, 6.5, 9.0, 11.5, and 14.0% of the total mass of vegetable spreads. The sample without these additions was used as the control for the experimental variants. The tested additions were selected based on a preliminary sensory analysis of the spreads.

The vegetable spreads were portioned (100 g) in jars (150 mL), screwed with a twist cap and thermally treated at 100 °C for 20 min using the CCC series convection oven (Rational, Landsberg am Lech, Germany) with 100% steam program. Then, the jars were cooled to room temperature and stored in a refrigerator at 4 °C, not longer than 1 week before analysis.

### 2.3. Chemical Composition and pH Analysis

The chemical composition of the fermented vegetable-based spreads was determined according to ISO standards, i.e., moisture [23], ash [24], protein [25] (Kjeltec-2200 system, Foss, Hilleroed, Denmark), fat [26] (Soxtec-HT6 system, Foss) content, and dietary fiber content according to the enzymatic-gravimetric AOCS method [27]. The results were expressed as g kg^−1^. The chemical composition analysis was performed in triplicate for each sample.

A portable S2-Food-Kit Seven2Go pH meter (Mettler Toledo) equipped with an InLab Solids Go-ISM electrode was used for pH evaluation. Five independent measurements were taken for each sample.

### 2.4. Phenolic Compound Analysis

Phenolic compound analysis was conducted by HPLC according to Tsao and Yang [28]. An Agilent 1260 Infinity HPLC system (Agilent Technologies, Palo Alto, CA, USA), equipped with Zorbax SBC-18, 5 µm, 4.6 x 150 mm column (Agilent Technologies, Palo Alto, CA, USA) was used. Ten grams of vegetable spread was weighed in a conical flask and 50 mL of aqueous methanol solution (70% *v*/*v*) was added. Then, it was homogenized for 1 min, shaken for 15 min and centrifuged at 5200 rpm for 15 min. The supernatant was collected, and the remaining residue was re-extracted in the same way with a new portion of solvent. Supernatants were combined, filtered through a vacuum filter, and evaporated at 45 °C. The evaporated sample was filled with distilled water to 25 mL and filtered through 0.45 μm PTFE filter. HPLC separation was conducted using solutions of 6% acetic acid in 2 mM sodium acetate (solvent A) and pure acetonitrile (solvent B). The flow rate was 1 mL min^−1^. The phenolic compound analysis was performed in triplicate for each sample.

Quantitative analysis of phenolic compounds was carried out using an external standard method. Hydroxybenzoic and hydroxycinnamic acids were quantified as syringic and coumaric, respectively, and flavonoids as quercetin. Qualitative analysis was performed based on the spectral properties of the following standards: syringic acid, coumaric acid, caffeic acid, ferulic acid, isoorientin, luteolin and apigenin (Sigma-Aldrich Chemie GmbH, Hamburg, Germany), and by comparison with the UV-Vis spectra according to references [29].

### 2.5. Antioxidant Activity Analysis

The antioxidant activity was determined using ABTS [30] and FRAP [31] assays. The extracts for phenolic compound analysis were used for antioxidant activity evaluation. The analyses were performed in triplicate for each sample.

For ABTS assay, appropriate dilutions of sample were added to chemically generated ABTS cation in PBS pH 7.4 buffer, mixed, and, after six-minute incubation at 30 °C, absorbance was read at 734 nm. Results were expressed as μM Trolox equivalent g^−1^ fresh mass (f. m.).

For FRAP assay, appropriate dilutions of sample were added to working FRAP reagent obtained by mixing acetate buffer (pH 3.6), tripyridyltriazine (TPTZ) solution and iron (III) chloride hexahydrate (FeCl_3_·6H_2_O) solution. Then, the solution was mixed and, after four-minute incubation at 37 °C, absorbance was read at 593 nm. Results were expressed as mM Fe^2+^ g^−1^ f. m.

### 2.6. Color and Viscosity Instrumental Analysis

The color of the vegetable spreads was determined using the Konica Minolta 3600 d spectrophotometer (Konica-Minolta, Osaka, Japan). Samples were directly put to the spectrophotometer aperture with bottom of the jars. Measurements were taken in six different areas in reflectance mode. Specular reflectance was excluded, illuminant D65 and 2° observation angle were used. Based on spectral reflectance measurement, color coordinates of CIE L*a*b* system were calculated. The mean values of L*, a* and b* were used for total color differences *dE* calculation according to the formula:

(1) $$dE=\sqrt{{\left(L_{0}^{*}-L_{s}^{*}\right)}^{2}+{\left(a_{0}^{*}-a_{s}^{*}\right)}^{2}+{\left(b_{0}^{*}-b_{s}^{*}\right)}^{2}}$$

where $L_{0}^{*}$, $a_{0}^{*}$, $b_{0}^{*}$ are color coordinates of control sample and $L_{s}^{*}$, $a_{s}^{*}$, $b_{s}^{*}$ are color coordinates of tested sample.

ViscoQC 300 (Anton Paar Gmbh, Graz, Austria) rotational viscometer was applied for viscosity analysis. The tests were performed at room temperature without additional adjustments. The samples were placed into a plastic vial c.a. 50 mL. For the measurements, dedicated L3 and L4 spindles were applied. The speed was adjusted up to the viscosity limits. Each test was performed in triplicate.

### 2.7. Microstructure Analysis

Morphology of the spread samples was evaluated using two types of optical microscopes. For the lower magnifications, a stereo microscope ZEISS Stemi 305 (Zeiss, Shanghai, China), equipped with a digital color camera Olympus, EP50 was used. Detailed morphology studies were conducted with an inverted microscope ZEISS AxioVert.A1 (Zeiss, Shanghai, China) and camera Axiocam 208 (Zeiss, China).

The samples were placed directly on the microscopic glass without additional preparation. The samples with low viscosity were pre-tested and imagined in ibidi slides or chambers (Gräfelfing, Germany). For the result presentation, the resolution of the images was adjusted using ZEN3.1 software (Zeiss, Jena, Germany).

### 2.8. Sensory Analysis

Sensory analysis was carried out using consumer analysis and sensory profiling methods. Analyses were conducted in the sensory analysis laboratory [32] at the Department of Gastronomy Science and Functional Foods, Poznan University of Life Sciences, Poland. Samples (15 g) were served in plastic containers (200 mL) with lids. The samples were coded with three-digit numbers, and the serving order was randomized (the program ANALSENS—v.5.0; Sopot; Poland). Unsweetened black tea (≈45 °C) was also served to neutralize the sample taste.

No ethical approval was required for the study. This study was conducted in accordance with the 1975 Helsinki declaration, which was revised in 2013. Participants were informed about the study’s aim and that participation was voluntary. They could stop the analysis at any point, and the responses would be anonymous. The authors did not ask for sensitive data or personal information. Formal dependence was not used to recruit subjects for the study. All subjects gave written informed consent to participate in the analysis.

The consumer analysis [33] was carried out with a group of 62 people aged 20–35. Women constituted 59% of the population analyzed. Consumers tested color, taste, aroma, consistency, and overall desirability. A 10 cm hedonic graphic scale was used, with the following margin denotations: “undesirable” to “highly desirable”. All consumers rated all samples in one session (order of administration: six samples, 0.5 h interval, and then five samples).

A 7-member tested panel participated in the sensory profiling analysis of products [34]. For the description of the product, four color descriptors (green, white, yellow, and grey), eight taste descriptors (pickled cucumber, vegetable, tart, sweet, bitter, sour, metallic, and strange) and three consistency descriptors (uniformity, spreadability, and firmness) were used. The intensity of each descriptor was determined using a 10 cm linear scale with the appropriate margin descriptions “undetectable” to “very intensive”.

### 2.9. Statistical Analysis

All experimental results were expressed as the mean ± standard deviation. The Statistica software (v.13.3. Tibco Software Inc., Palo Alto, CA, USA) was used for statistical analyses. The results were subjected to the analysis of variance (ANOVA), and then post hoc Tukey’s test was applied (*p* < 0.05) to compare the means. The Pearson correlation coefficient (*r*) and determination coefficient (*r*^2^) were computed to assess the relationships between the variables.

## 3. Results and Discussion

### 3.1. Effect of Chia and Flaxseed Meal on pH Changes and Chemical Composition of the Vegetable Spreads

Introducing chia and flaxseed meal into the fermented vegetable-based spreads changed their pH values (Table 2). The control sample (without meal addition) had a pH value of 3.5. The pH value of the spreads significantly increased with the increase in meal contents and were 4.23 and 4.47 for the samples with the highest chia and flaxseed meal additions (C 14% and F 14%), respectively. The pH value increase was higher in the case of flaxseed meal addition than in the case of chia. Other authors also concluded that adding 5% flaxseed powder to yoghurt increased the pH from about 4.0 to 4.3 [35], and chia seed introduction increased the pH from 4.2 to 4.3 [36]. Increasing the pH value could affect the taste of vegetable spread with meal addition. Food with lower pH could present a tart and sour taste, whereas those with higher pH values may have a more subdued profile.

The addition of chia and flaxseed meal considerably influenced the chemical composition of the fermented vegetable-based spreads (Table 2). The chemical composition of the control sample was as follows (per kg): 0.03 g of fat, 1.35 g of protein, 1.91 g of ash, and 2.87 g of total dietary fiber. The fat, protein, ash, and dietary fiber content of the spreads significantly increased with the increase in meal additions. The samples with the highest chia and flaxseed meal additions (C 14% and F 14%) had 41.96 g kg^−1^ and 8.33 g kg^−1^ of fat and 32.28 g kg^−1^ and 44.30 g kg^−1^ of protein, respectively. The difference in the contents related to the meal compositions were declared by the producer, i.e., higher fat and lower protein content in the chia seeds than defatted flaxseed meal. The dietary fiber (58.76 g kg^−1^ and 54.41 g kg^−1^) and the ash (17.07 g kg^−1^ and 17.78 g kg^−1^) contents were similar in the vegetable spreads with the 14% addition of chia and flaxseed meal.

**Figure 1:**
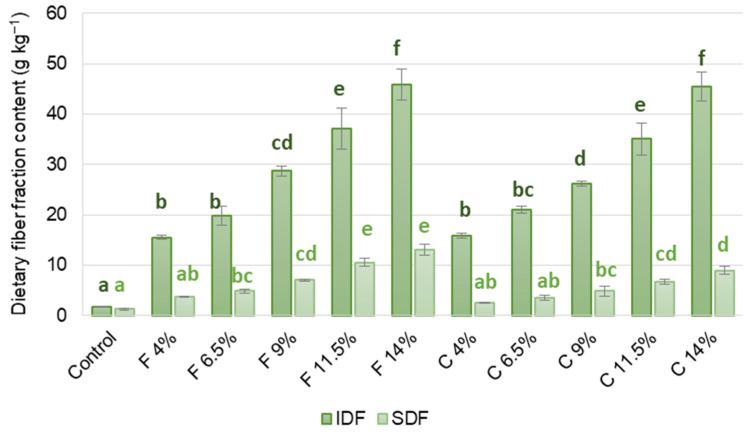
Soluble (SDF) and insoluble dietary fiber (IDF) content in the fermented vegeta-ble-based spreads with various chia or flaxseed meal additions. Sample codes: as in Table 2. Error bars are the standard deviation of means. For each dietary fiber fraction, bars marked with different letters (dark green—IDF, light green—SDF) are significant-ly different (one-way ANOVA, Tukey HSD test at α = 0.05).

The analysis of soluble (SDF) and insoluble dietary fiber (IDF) fractions (Figure 1) in the vegetable spreads with the chia and flaxseed meal additions showed similarities in the IDF content for the samples with the same addition amount. However, the higher SDF content was in those with flaxseed meal addition (where it consists of about 20% of total dietary fiber—TDF) than chia seed (consisting of about 15% of TDF). Kosiorowska et al. [37] also reported that adding flaxseed meal or chia seed to cranberry jam resulted in a higher share of soluble dietary fiber (SDF) in the flaxseed-added product (58% TDF) compared to the chia-added product (40% TDF). The difference in SDF content relates to the SDF: IDF ratio characterizing dietary fiber in chia and flax seeds. The SDF to IDF ratio is between 20:80 and 40:60 in flax seeds [38] and 16:84 in chia seeds [39]. The differences may be crucial for the ability of the applied meal to modify the designed vegetable spread quality.

Our study showed that both chia and flaxseed meal improve the nutritional composition of the designed fermented vegetable-based spreads significantly increasing fat, protein, and dietary fiber contents. The increase in protein content is so high that it allows the use of nutrition claims such as “source of protein”/ or “high protein” for the product according to European Union regulations (Regulation (EU) No 1047/2012 [40]). The claims “source of protein” (= “at least 12% of the energy value of the food is provided by protein”) and “high protein” (= “at least 20% of the energy value of the food is provided by protein”) can be applied for all spreads with chia seed meal addition and all spreads with flaxseed meal addition, respectively, regardless of the amount of added meal. The energy content calculation (based on the spread composition shown in Table 1 and standardized energy conversion factors recommended by FAO, [41]) shows that 14–17% of the energy value of the spreads with chia seed meal and 21–31% of the energy value of the spreads with flaxseed meal is provided by protein. Moreover, the vegetable spreads with 9% of meal and higher, regardless of the meal type, characterized as high dietary fiber content (between 3.5 g and 5.9 g of fiber 100 g^−1^) meet EU requirements by using the nutrition claim “source of fiber” (i.e., “the product contains at least 3 g of fiber per 100 g”). These nutrition claims can be valuable product attributes for customers and encourage them to purchase.

### 3.2. Effect of Chia and Flaxseed Meal on Phenolic Composition and Antioxidant Activity of the Vegetable Spreads

In our study, the applied procedure allowed for identifying and quantifying only free phenolic compounds present in the samples (i.e., those not bound by ester-linkages).

For the control sample, the total determined phenolic compound content was 2.70 mg 100 g^−1^ (Table 3). Derivatives of syringic acid, coumaric acid, and caffeic acid were identified in the profile, with syringic acid being the predominant compound. Ciniviz and Yildiz [42], in their study on the phenolic content of lacto-fermented cucumber pulp, also identified syringic acid as the dominant compound, with a concentration of 4.59 mg 100 g^−1^. Direct studies on fermented zucchini are scarce. The predominance of syringic acid in fresh zucchini species was noted by Stryjecka et al. [43]. Inswaldi et al. [44] and Abd-Elkader at al. [45] also detected (HPLC and HPLC-MS analysis) syringic, *p*-coumaric and caffeic acid among other phenolic compounds in fresh zucchini species.

The chia seed meal and flaxseed meal addition enriched the fermented vegetable-based spreads in phenolic compounds. The 4% addition of flaxseed meal resulted in a significant increase in phenolic compounds content (Table 3). In the phenolic profile, vanillic acid, isoorientin, and luteolin derivatives were identified. A significant increase in coumaric acid derivatives was also found. The highest concentrations of phenolic compounds (ranging from 7.60 to 8.65 mg 100 g^−1^) were found in the F 9%, F 11.5%, and F 14% samples, with a significant increase in the levels of syringic acid, vanillic acid, caffeic acid, and flavonoids including isoorientin and luteolin derivatives. Additionally, apigenin was detected in the profile. Gai et al. [46] identified derivatives of coumaric and caffeic acids, as well as apigenin, luteolin, coniferyl alcohol β-D-glucoside, and dehydrodiconiferyl alcohol-4-O-glucoside in flax seeds. Kaur et al. [47] reported the presence of vanillic acid and syringic acid, along with chlorogenic acid, gallic acid, ferulic acid, protocatechuic acid, catechin, epicatechin, rutin, and quercetin. The literature data indicate that the predominant phenolic fraction in flaxseeds consists of lignans, particularly secoisolariciresinol diglucoside (SDG) [19,48]. However, SDG forms macromolecular complexes in the seeds, which include, among others, diglucoside herbacetin, as well as derivatives of coumaric acid, caffeic acid, and ferulic acid ester-linked to hydroxymethylglutaryl groups [49,50]. Consequently, in the analysis of free phenolic compounds, without hydrolysis of the ester bonds, SDG and some bounded compounds associated within this macromolecular complex could remain undetectable.

In the case of samples with the addition of chia seed meal (samples C 4–C 14%), a significant increase in coumaric acid derivatives and the phenolic profile enrichment with rosmarinic acid, ferulic acid, and quercetin was observed (Table 3). Among the phenolic compounds derived from chia seeds, rosmarinic acid was dominant. A similar phenolic profile in chia seed meal was also reported by Rahman et al. [18] and Gebremeskal et al. [51]. Rahman et al. [18] additionally identified protocatechuic acid, caffeic acid, myricetin, dihydroxyisorhamnetins, and daidzein in the free phenolic fraction. In the fraction obtained after hydrolysis, they also detected the presence of quercetin, rutin, apigenin, and procyanidins. The differences in the profiles of the detected compounds may be attributed to variations in the growing conditions [52]. With the addition of 6.5% and 9% chia seed meal to the spreads, a significant increase in the phenolic content was recorded (by about 70% and 130% of the control, respectively). The highest content of the compounds was found in the C 11.5% and C 14% samples. The increasing meal proportion in the spreads primarily led to higher concentrations of rosmarinic and ferulic acids. Comparing the tested samples, it can be observed that the samples with chia seed meal had a higher free phenolic compound content than those with flaxseed meal. However, no significant differences were found between the 11.5% and 14% addition of particular meal.

**Figure 2:**
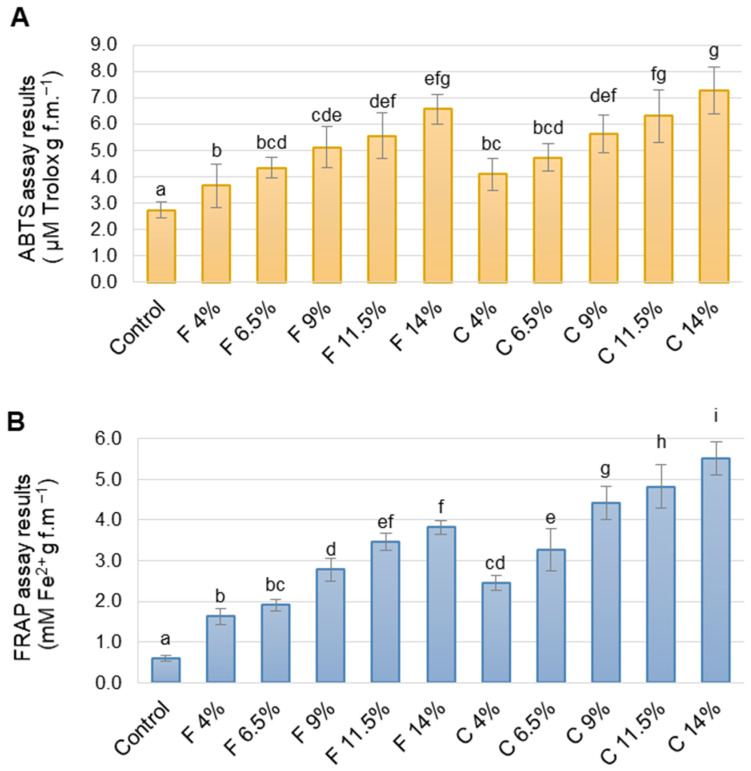
Antioxidant activity of the fermented vegetable-based spreads with various chia or flaxseed meal additions. (**A**) ABTS assay results (**B**) FRAP assay result. Sample codes: as in Table 2. Error bars are the standard deviation of means. Bars marked with different letters are significantly different (one-way ANOVA, Tukey test at α = 0.05).

The antioxidant capacity of the fermented vegetable-based spreads evaluated using ABTS and FRAP assay are shown in Figure 2.

The antioxidant activity of the spread composed only from fermented zucchini and cucumber (the control) were 2.76 ± 0.33 μM Trolox g^−1^ and 0.60 ± 0.07 mM Fe^2+^ g^−1^ as determined by ABTS and FRAP assay, respectively. The value range obtained with ABTS assay was different than FRAP and the differences could be caused by different reaction mechanism of the assays.

The addition of chia or flaxseed meal caused a considerable increase in the sample’s antioxidant activities. The scope of changes depended on the meal added and the assay used, but showed a similar tendency, regardless of the methods (ABTS or FRAP). For the spread with 4% chia seed meal addition (C 4%), the ABTS value increased by about 50%, and FRAP value was about 300% higher than the control. For flaxseed meal addition, the results of F 4% samples were 40% and 170% higher than the control for ABTS and FRAP results, respectively. The antioxidant activity increased when the meal addition was increased regardless of the meal type and measurement method, and the changes presented a linear fit. The determination coefficient (*r*^2^) for this regression model was 0.98 or higher.

The results showed that incorporating chia or flaxseed meal into vegetable spreads could be a good way to increase the antioxidant properties of food products. Pająk et al. [53] tested the antioxidant properties of chia and golden flax seeds. Their research demonstrated that chia seeds had higher antioxidant activity than flax seeds. Chia and flax seeds are renowned for their rich antioxidant properties, primarily due to the high content of phenolic compounds. In our study, ABTS assay results (*r* =0.940 and 0.949 for chia and flaxseed meal, respectively) and FRAP one (*r* = 0.984 and 0.977) strongly correlated with the total phenolic compound content. This indicates that phenolic compounds appear to be the primary contributors to the antioxidant activity of the spread samples.

### 3.3. Effect of Chia and Flaxseed Meal on Total Color Differences (dE) and Viscosity Parameters of the Vegetable Spreads

The total color difference values (*dE*) of the vegetable spread with chia or flaxseed meal additions were calculated compared to the control sample (Table 4).

The *dE* values were between 3.8 and 14.9, and the differences were visually recognizable by the observer (*dE* > 3.5; according to Mokrzycki and Tatol assay [54]). For most samples, the meal caused such a high difference in the sample color from the control (*dE* > 5) that they could be registered as two different colors. The flaxseed meal addition to the vegetable spreads caused higher differences in the color than the chia seed meal one, and they increased with the increasing meal addition (the highest value for the F14% sample). The color became brighter and more yellow due to the golden-seed flax with yellow seed husk used in the study. An increase in brightness and yellowness of the color due to the ground flaxseed addition to gooseberry and cranberry jams was also noted by Banaś et al. [55] and Kosiorowska et al. [37]. In the case of samples with chia seed meal, the highest *dE* was the C9% sample, and the further increase in the meal addition made the spread color darker due to the more visible black seed husk.

Most food spreads are composed from fruits, dairy products, edible oils, or nut butters [56]. Alternatively, cocoa-based products are most desirable to children. One of the keys to the success of spread formulation is that they are easy to use and adhere to bread, which is relevant to viscosity. Moreover, the consumers do not prefer the food structures for the bread spreading, which contain a high amount of water (typically with high water content).

In the study, all tested vegetable-based spreads behaved like a non-Newtonian fluid. The control samples characterized the lowest viscosity η = 3.77 Pa·s related to the high water content in the product.

The addition of chia and flaxseed meal influenced the spread viscosity (Table 4). The dynamic viscosity increased from 14.6 to 391 Pa·s for the samples with chia seed meal and from 14.6 to 306 Pa·s for those with flaxseed meal, respectively. However, the increase in the dynamic viscosity was not linear. Moreover, the effect of the meal type on the spread viscosity differed depending on the meal addition. The lower level of chia or flaxseed meal in spreads (4% and 6.5%) modified the viscosity similarly. For the high chia seed meal additions, a rapid increase (approximately 15-fold) in viscosity was for C 9% samples, and the further increase in meal amount did not significantly change the viscosity. A similar rapid increase in dynamic viscosity of the samples with flaxseed meal was observed only for the F 14% samples.

The increase in the viscosity of the spreads with chia or flaxseed meal probably resulted from the increased water-soluble dietary fiber (SDF) and proteins introduced into spreads by the additions. These compounds exhibit a high water-binding capacity and gelling properties [7,9]. In our study, the statistical analysis showed a high and significant linear correlation between SDF content and the dynamic viscosity of spreads (*r* = 0.849 and 0.845 for the samples with chia and flaxseed meal, respectively). It confirms our assumption concerning the SDF. Such reported SDF functional properties, such as water-holding capacity, swelling capacity, and the ability to form viscous gels [3,57], which reveal the interaction between SDF and water, could be crucial for improving the consistency of the fermented vegetable-based spread. The study by Kosiorowska et al. [37] on the effect of whole or ground flax and chia seeds on the physicochemical properties of cranberry jams also showed the seed gelling properties and the correlation between the dietary fiber content and the hardness parameters of jams.

The difference between the viscosity of spreads with a higher addition of chia and flaxseed meal addition may be related to the difference in gelling properties. It has been shown that the soluble fibers released into water by the chia seeds form hydrogel structures at pH 3 to 12 [58]. For flaxseed soluble fiber (found in mucilage), the rheological properties of water suspension depend on the fiber composition, i.e., neutral to acidic polysaccharide ratio—they may form a weak gel with a higher neutral polysaccharide amount but appear as a viscoelastic fluid at high acidic polysaccharides share [7]. Viscosity or fluidity of flaxseed dietary fiber suspensions are also affected by pH (the viscosity increases by increasing pH from 2 to 8).

The observed eye consistency of the spread samples with the highest addition of chia and flaxseed meal is also worth noting. The spread samples were easy to detach from the walls of the plastic test tubes. It may be crucial for packed planning in the future, e.g., in plastic packaging.

### 3.4. Effect of Chia and Flaxseed Meal on Microstructure of the Vegtable Spreads

**Figure 3:**
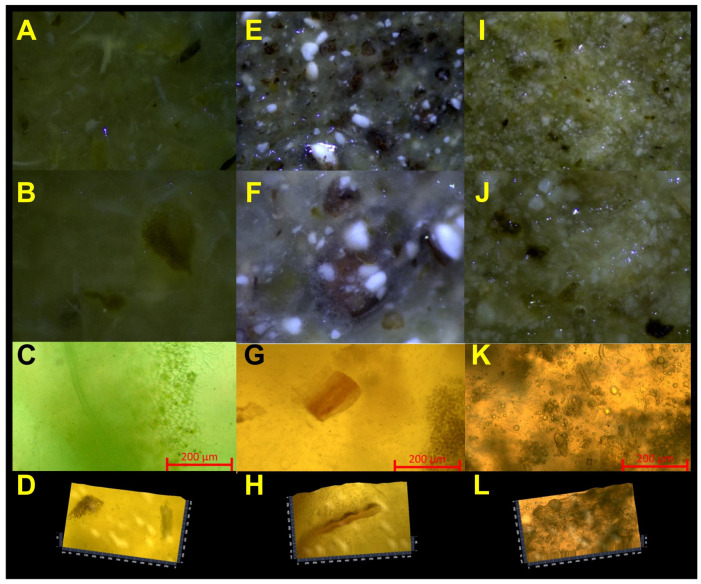
Microscopic images of the fermented vegetable-based spreads: Control ((**A**,**B**) stereoscopic microscope, (**C**) inverted microscope, (**D**) inverted microscope 2.5D image); C 9% sample ((**E**,**F**) stereoscopic microscope, (**G**) inverted microscope, (**H**) inverted microscope 2.5D image); and F 9% sample ((**I**,**J**) stereoscopic microscope, (**K**) inverted microscope, (**L**) inverted microscope 2.5D image).

In our study, the rheological and sensory tests were supported with microstructure analysis performed by optical microscopes. The image examples are shown in Figure 3.

High non-homogeneity was observed in all samples. Moreover, the mucus was visible, especially in the samples with chia seed meal addition (images from a stereoscopic microscope—Figure 3E,F). More detailed microscopic analysis using an inverted microscope also indicated the presence of fibers in the samples. The microscopic examinations showed complete or partial disintegration of chia seeds under grinding. In the case of the samples with flaxseed meal, the images of white structures with an oval or irregular shape with a slightly hair-like structure could be distinguished and resulted from the meal addition.

All images taken with a higher magnification and using an inverted microscope also showed the presence of oil drops. The irregular shape of the vegetables observed in higher magnification was related to the technique of vegetable grinding.

In our previous studies [59], we proposed a stepwise path to study the structure of foods according to their size and homogeneity. Here, differences between the spread samples were observed using a simple stereoscopic microscope. The previous De Oliveira et al. study was focused mainly on microscopic food control due to possible contamination [60]. However, this research showed that the spread preparation process may also be controlled this way.

### 3.5. Effect of Chia and Flaxseed Meal on Sensory Quality and Consumer Acceptance of the Vegetable Spreads

**Figure 4:**
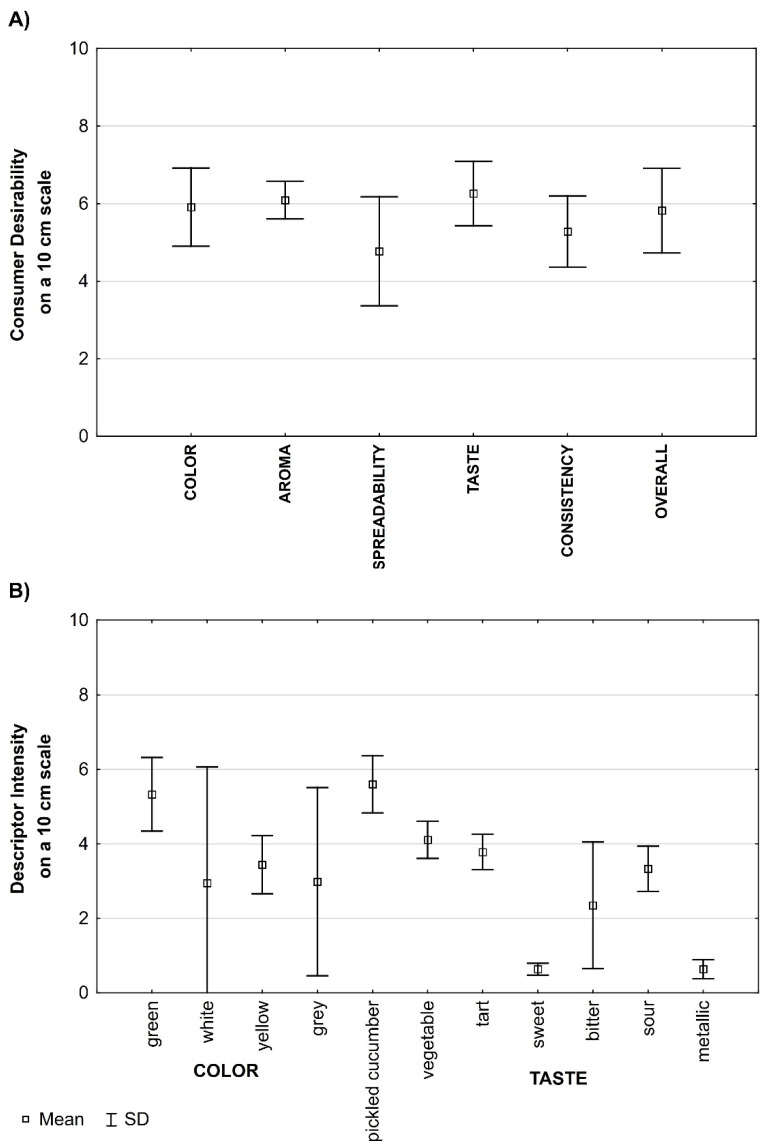
Sensory evaluation of the fermented vegetable-based spreads with various chia or flaxseed meal additions. (**A**) Box plot diagram of product attribute desirability scores in consumer analysis (**B**) Box plot diagram of the color, taste, and consistency descriptor intensity in sensory profiling analysis.

Sensory quality is one of the most important determinants among the factors influencing consumer purchase behavior in the food market. Consumer analysis proved that the high overall desirability of most tested fermented vegetable-based spreads (Figure 4A). The values ranged from 5.00 to 7.60 points (Table S1, Supplementary Materials). The highest overall desirability scores were for the samples with flaxseed meal addition ≥ 9%.

The variant of addition (chia or flaxseed meal at various amounts) did not significantly affect taste desirability (*p* > 0.05). However, significant differences were found in consumers’ ratings for spreadability and color. In their opinion, the flaxseed meal additions improved the spreads’ spreadability. For spreadability, significantly higher desirability scores of F 11.5% and F 14% (6.5 points and 7.8 points, respectively) were noted compared to the control (3.0 points). These spread samples also had significantly higher scores of color desirability (7.50 and 8.00 points, respectively) than the control (5.40 points).

For the tested spreads, sensory profiling analysis defined and determined perception of the following descriptors: pickled cucumber, vegetable, tart, sweet, bitter, sour, metallic, and strange for taste; uniformity, spreadability, and firmness for consistency; green, white, yellow, and grey for color. The statistically significant differences were among sample profilographs (i.e., descriptors’ type and intensity in the sensory profiling analysis) depending on the meal type and amount added (Tables S2 and S3, Supplementary Materials).

For the taste descriptors, the highest variability in the results was for the bitter descriptor (0.5–3.2 points), pickled cucumber (4.3–6.8 points), sour (1.7–4.2 points), and tart (2.5–4.2 points) (Figure 4B). The statistical analysis (Pearson correlation coefficient) showed a negative correlation between the taste desirability and tart taste (*r =* −0.77), sour taste (*r* = −0.755) or bitter taste (*r =* −0.47) intensity (Table 5). The meal addition influenced the bitter taste intensity of the spreads (Table S2). The addition of chia seed meal increased the bitter taste intensity. The intensity was 3.00–3.20 points for C 11.5% and C 14% samples, compared to 1.0 points for control. On the contrary, the lowest bitter taste intensity was found in the samples with the highest flaxseed meal addition, i.e., F 11.5% and F 14% (0.5 points). The presence of both meals in the spreads decreased the tart taste intensity, and the flaxseed meal addition also decreased the sour taste intensity. The tastes also negatively correlated with the spread’s overall desirability (Table 5).

Statistical analysis confirmed a high positive correlation between consumer overall desirability and consistency (*r* = 0.82) or spreadability (*r* = 0.77) desirability of the tested spreads. A positive correlation was also found between the spreadability desirability and intensity (*r* = 0.89). The samples with lower desirability of spreadability (i.e., control, F 4% and C 4%) had lower uniformity and spreadability scores in the profiling analysis (Table S2). The increase in chia or flaxseed meal additions (≥6.5%) increased the spreadability scores of the vegetable spreads; the highest had F 11% and F 14% samples. However, there was no repeated correlation between the intensity value of firmness and uniformity descriptors and consumer analysis (*r* = 0.27 and *r* = 0.33, respectively; Table 5).

For color profiling descriptors (Table 5), the positive correlation was between the intensity of green color descriptors and consumer analysis parameters (*r* = 0.93). It also confirmed a negative correlation between the intensity of yellow (*r* = −0.61),white (*r* = −0.48) and grey color descriptors (*r* = −0.45) and consumer color desirability. It suggests that maintaining the green shade of color for the cucumber-zucchini spread was crucial for meeting consumer expectations. The chia seed meal addition influenced the spread color (due to the incorporation of dark color from black seed husk) that negatively impacted consumer color desirability compared to the spreads with gold (yellow) flaxseed meal, especially for higher meal additions.

Summarizing the sensory analysis results, samples with flaxseed meal addition ≥ 9% were highly desirable, particularly for their color and spreadability attributes. In consumer opinion, it was higher than the control sample (without the meal addition). The results related to the decreased intensity of bitter, tart, and sour tastes (i.e., those negatively correlated with product consumer desirability) and the increase in the feeling of spreadability intensity. Moreover, the flaxseed meal (from yellow seed in the research) improved the color desirability of the tested vegetable-based spreads. Chia seed meal addition improved the feelings of spread uniformity and spreadability, as did the flaxseed meal. Contrary to the flaxseed meal, it did not influence the sour taste intensity of the fermented vegetable-based spreads and introduced a bitter taste into the product taste. The samples with chia seed meal addition (≥6.5%) had a higher bitter taste intensity than the control, and a higher sour taste intensity than those with flaxseed meal. The increased intensity of the bitter taste may be related to the presence of phenolic compounds characteristic of chia seeds introduced to the fermented vegetable-based spreads with their addition. For example, quercetin and luteolin derivatives identified in the phenolic compound profile of the spreads with chia seed and flaxseed meal, respectively (Table 3), are classified as “bitter compounds” [61]. However, their share was low in the phenolic profiles, and the increase in product bitterness was only in the case of chia seed meal addition and not for flaxseed meal. Therefore, it is a syntax for supposing that the increase in the bitter taste of spreads with high chia seed meal addition may explain the interaction or synergism of chia seed compounds with other bitter compounds present in fermented vegetables (e.g., bitter peptides) or the formation of new compounds during spread production (e.g., at low pH or lipid oxidation conditions). The research of Gläser et al. [62] showed a significant bitter taste contribution of lipids and their oxidation products in pea-protein isolates (i.e., free fatty acids—α-linolenic acid and linoleic and trihydroxy octadecenoic acids—were key inducers of bitterness). Previous studies reported the increased intensity of bitter flavors in the sensory profile of products with chia seeds; however, it concerned low-pH products (e.g., yoghurt) or those with the addition of fermented chia seeds. Nakov et al. [36] reported that the yoghurt fortification with chia seeds caused a bitter aftertaste of the product, increasing with higher chia seed concentrations (10% vs 5% addition) and negatively affecting the overall sensory acceptance of the product among consumers. The authors attributed this to the degradation of the chia seed compounds and their interaction with the yoghurt matrix, and they pointed out that lipid oxidation and microbial activity could also contribute to these changes. Bartkiene et al. [63] studied the effect of fermented chia seeds (solid-state and submerged fermentation with *Lactiplantibacillus plantarum*; addition between 10% and 30%) on wheat bread sensory profile, and they noted higher bitterness for bread with fermented chia seeds (>10% addition) compared to the control without the addition, and no increase in bitter flavor even within the highest concentration of unfermented chia seeds in the product. On the other hand, the increase in bitterness of the bread with the addition of ground flaxseed cake fermented with *Lactiplantibacillus plantarum* was not observed [64]. Further research is needed to elucidate a bitter taste formation in fermented vegetable-based spreads upon chia seed meal addition.

There is a lack of studies on designing fermented vegetable-based food with chia and flaxseed meal to compare our results of sensory analysis. However, these seeds were used as ingredients in fruit preserves, i.e., jams [37,55,65]. Banaś and co-authors [55] reported that the low-sugar gooseberry jams with ground defatted flax seeds added (3%) received high scores (4.5 on a 5-point scale) during sensory evaluation. These results are similar to our own. Nduko et al. [65] studied the consumer acceptance of pineapple jams fortified with whole chia seeds depending on their addition (6.25–50%). They reported that 6.25% chia seed addition did not vary the color and texture of the product significantly compared to the control, but their higher amounts (≥12.5%) decreased the attribute scores. On the other hand, the jam spreadability was similar, even for the highest chia seed addition. The authors explained that the low texture of jams at higher concentrations of chia seeds by the use whole chia seeds in their study and the contribution of chia seed gums to jam gelation but not affecting the spreadability. Our study showed higher scores for the spreadability and desirability of the product with chia seed meal (but not statistically significant, Table S1), despite the significantly higher spreadability intensity than the control (sensory profiling, Table S2), which can be explained by the use of ground chia seeds (meal) in our study and the contribution of both chia seed mucilage and proteins extracted from the meal on the product sensory quality.

In our study, we also analyzed the linear correlation between consumer analysis of the product attributes (consumer desirability) and the parameters of instrumental analysis (i.e., viscosity and total color difference *dE* values; Section 3.3). A statistically significant (*p* < 0.05) and positive correlation was found between the *dE* values and consumer color desirability (*r* = 0.618), as well as *dE* values and overall desirability (*r* = 0.795)—the higher *dE* values, higher consumer desirability of the vegetable spread variant. Correlations between viscosity value and consumer spreadability desirability (*r* = 0.461), consistency desirability (*r* = 0.174) or overall desirability (*r* = 0.045) were not statistically significant (*p* > 0.05). However, when the samples were grouped according to a meal type, correlations between the sample viscosity and spreadability desirability were statistically significant, and Pearson correlation coefficients were *r* = 0.891 for the chia seed meal addition and *r* = 0.902 for flaxseed meal addition, respectively. The results indicate that these instrumental parameters can be applied to predict the direction of consumer desirability of the newly designed fermented vegetable-based spreads.

## 4. Conclusions

Our research showed that chia and flaxseed meal can be applied as an ingredient of fermented vegetable-based spreads to design their nutritional composition and sensory quality.

Chia or flaxseed meal additions improve the nutritional composition of the spreads compared to one without the addition, including protein and dietary fiber content. Moreover, introducing chia or flaxseed meal into fermented zucchini-cucumber spread considerably extends the vegetable spread phenolic profile and increases its antioxidant capacity. However, only flaxseed meal additions can efficiently improve the product’s sensory quality. This ingredient efficiently masks the intensity of undesirable fermented vegetable flavors such as bitter, tart, and sour tastes, and improves spreadability, consistency, and uniformity. It increases the overall consumer desirability of the fermented vegetable-based spreads. The chia seed meal also corrects the uniformity and spreadability of the spread but does not influence the sour taste intensity. The main problem with chia seed application is a perceptible increase in a bitter taste. The understanding of a bitter taste formation mechanism in the spreads when adding chia seed meal should be considered in future studies.

The results of this study showed that the spreadability and color desirability of the fermented vegetable-based spreads were significantly related to changes in the product viscosity and color due to the meal additions recorded by instrumental analysis, respectively. It suggests that instrumental methods may be applied to predict the direction of consumer desirability of the newly designed foods. However, the issue needs further research.

Based on the results, the flaxseed meal additions between 9% and 11.5% are recommended as ingredients of the newly designed fermented vegetable-based spreads. Such products can be a new healthy alternative to the other non-vegetable spreads on the food market. The target of the spreads could be vegan consumers or those with lactose intolerance (who can consume them as dairy product substitutes). They could also be alternatives to high-fat spreads based on rich in saturated fatty acid ingredients. As dietary fiber source, the spreads could be attractive for diabetes or overweight people. It can be a new product line that increases the use of vegetables for food production, including those that do not meet consumer expectations for culinary use (e.g., overgrown, undergrown, externally rubbed but not spoiled). Further research could be expedient for testing other compositions of fermented vegetables as a base for spreads, particularly to extend the sensory attributes of the final product.

## Figures and Tables

**Table 1:** Formulation of vegetable spreads (g kg^−1^).

Components	Spread Samples					
	Control	4%	6.5%	9%	11.5%	14%
Fermented cucumbers	500	480	467.5	455	442.5	430
Fermented zucchini	500	480	467.5	455	442.5	430
Chia/Flaxseed meal	-	40	65	90	115	140

**Table 2:** Chemical composition and pH value of the fermented vegetable-based spreads with various chia or flaxseed meal additions.

Sample	Chemical Composition (g kg^−1^)					pH
	Moisture	Fat	Protein	Ash	Dietary Fiber	
Control	961.2 ± 2.5 ^g^	0.030 ± 0.002 ^a^	1.35 ± 0.11 ^a^	1.91 ± 0.16 ^a^	2.87 ± 0.06 ^a^	3.51 ± 0.02 ^a^
Flaxseed meal						
F 4%	918.2 ± 1.8 ^f^	2.72 ± 0.08 ^b^	13.74 ± 0.75 ^c^	9.79 ± 0.83 ^b^	19.11 ± 0.51 ^b^	3.80 ± 0.02 ^c^
F 6.5%	896.7 ± 3.6 ^e^	4.14 ± 0.21 ^bc^	20.26 ± 1.09 ^d^	11.41 ± 1.47 ^bc^	24.61 ± 1.88 ^b^	3.94 ± 0.01 ^e^
F 9%	872.4 ± 1.0 ^c^	5.76 ± 0.07 ^d^	27.45 ± 0.01 ^e^	14.08 ± 0.45 ^d^	35.66 ± 1.28 ^cd^	4.10 ± 0.01 ^g^
F 11.5%	847.1 ± 6.2 ^b^	7.76 ± 0.06 ^e^	36.05 ± 2.31 ^g^	14.74 ± 1.37 ^d^	47.51 ± 3.96 ^e^	4.25 ± 0.01 ^h^
F 14%	825.3 ± 6.0 ^a^	8.33 ± 0.23 ^f^	44.30 ± 0.98 ^h^	17.07 ± 0.34 ^e^	58.76 ± 2.14 ^f^	4.47 ± 0.02 ^i^
Chia seed meal						
C 4%	925.4 ± 2.6 ^f^	5.31 ± 0.14 ^cd^	6.94 ± 0.26 ^b^	9.48 ± 0.27 ^b^	18.38 ± 0.54 ^b^	3.70 ± 0.01 ^b^
C 6.5%	901.9 ± 2.1 ^e^	8.33 ± 0.19 ^e^	11.95 ± 0.26 ^c^	13.24 ± 0.56 ^cd^	24.57 ± 1.18 ^b^	3.84 ± 0.01 ^d^
C 9%	884.5 ± 3.2 ^d^	12.32 ± 0.30 ^f^	14.46 ± 0.39 ^c^	9.21 ± 0.14 ^b^	31.01 ± 1.46 ^c^	3.97 ± 0.01 ^f^
C 11.5%	853.2 ± 5.3 ^b^	17.64 ± 1.57 ^g^	21.53 ± 1.05 ^d^	12.82 ± 0.97 ^cd^	41.73 ± 3.42 ^de^	4.08 ± 0.02 ^g^
C 14%	816.2 ± 4.9 ^a^	41.96 ± 0.65 ^h^	32.28 ± 0.44 ^f^	17.78 ± 0.74 ^e^	54.41 ± 3.17 ^f^	4.23 ± 0.01 ^h^

Sample codes: control—the samples without the meal addition; F 4%, F 6.5%, F 9%, F 11.5%, F 14% and C 4%, C 6.5%, C 9%, C 11.5%, C 14–the samples with 4, 6.5, 9, 11.5, and 14% addition of flaxseed meal (F) or chia seed meal (C), respectively. Mean ± SD. In each column, means marked with different superscript letters are significantly different (one-way ANOVA, Tukey HSD test at α = 0.05).

**Table 3:** Phenolic compound composition of the fermented vegetable-based spreads with various chia or flaxseed meal additions.

Samples	Phenolic Compound Content (mg 100 g^−1^)										
	Syringic Acid	Vanillic Acid	Coumaric Acid Derivatives	Caffeic Acid	Rosmarinic Acid	Ferulic Acid Derivatives	Isoorietin	Luteolin Derivatives	Apigenin	Quercetin	Total
Control	2.40 ± 0.07 ^b^	nd	0.11 ± 0.02 ^a^	0.19 ± 0.01 ^a^	nd	nd	nd	nd	nd	nd	2.70 ± 0.04 ^a^
F 4%	2.30 ± 0.14 ^ab^	0.09 ± 0.01 ^a^	1.27 ± 0.08 ^b^	0.45 ± 0.03 ^c^	nd	nd	0.19 ± 0.02 ^a^	0.17 ± 0.01 ^a^	nd	nd	4.46 ± 0.21 ^b^
F 6.5%	2.84 ± 0.03 ^c^	0.21 ± 0.01 ^b^	1.19 ± 0.02 ^b^	0.84 ± 0.03 ^d^	nd	nd	0.24 ± 0.05 ^a^	0.24 ± 0.05 ^a^	nd	nd	5.57 ± 0.01 ^bc^
F 9%	3.47 ± 0.01 ^d^	0.31 ± 0.01 ^c^	1.74 ± 0.06 ^b^	1.17 ± 0.11 ^e^	nd	nd	0.32 ± 0.01 ^b^	0.32 ± 0.01 ^b^	0.09 ± 0.01 ^a^	nd	7.60 ± 0.18 ^cd^
F 11.5%	3.47 ± 0.05 ^d^	0.41 ± 0.01 ^d^	2.16 ± 0.01 ^b^	0.66 ± 0.01 ^e^	nd	nd	0.33 ± 0.01 ^c^	0.33 ± 0.01 ^b^	0.09 ± 0.02 ^a^	nd	8.33 ± 0.10 ^d^
F 14%	3.66 ± 0.15 ^d^	0.45 ± 0.01 ^e^	2.24 ± 0.05 ^b^	1.16 ± 0.02 ^e^	nd	nd	0.38 ± 0.03 ^c^	0.38 ± 0,03 ^b^	0.09 ± 0.01 ^a^	nd	8.65 ± 0.31 ^d^
C 4%	2.00 ± 0.12 ^a^	nd	5.17 ± 0.06 ^c^	0.12 ± 0.02 ^ab^	1.52 ± 0.04 ^a^	6.3 ± 0.6 ^a^	nd	nd	nd	0.30 ± 0.02 ^a^	15.4 ± 0.6 ^e^
C 6.5%	2.24 ± 0.18 ^ab^	nd	9.75 ± 0.50 ^d^	0.13 ± 0.04 ^ab^	2.52 ± 0.06 ^b^	10.5 ± 0.4 ^b^	nd	nd	nd	0.46 ± 0.02 ^b^	26 ± 1 ^f^
C 9%	2.24 ± 0.08 ^ab^	nd	15 ± 1 ^e^	0.19 ± 0.06 ^b^	3.34 ± 0.03 ^c^	13.6 ± 0.2 ^c^	nd	nd	nd	0.60 ± 0.04 ^c^	35 ± 1 ^g^
C 11.5%	2.10 ± 0.08 ^ab^	nd	17 ± 1 ^e^	0.16 ± 0.01 ^ab^	3.77 ± 0.05 ^d^	14.6 ± 0.3 ^c^	nd	nd	nd	0.63 ± 0.01 ^c^	38 ± 1 ^h^
C 14%	2.18 ± 0.01 ^ab^	nd	17 ± 1 ^e^	0.17 ± 0.01 ^ab^	4.16 ± 0.12 ^e^	16.1 ± 0.7 ^d^	nd	nd	nd	nd	40.1 ± 0.1 ^h^

Sample codes: as in Table 2. Mean ± SD. In each column, means marked with different letters are significantly different (one-way ANOVA, Tukey HSD test at α = 0.05); nd—not detected.

**Table 4:** Changes in the total color differences (*dE*) and viscosity parameters of the fermented vegetable-based spreads with various chia or flaxseed meal additions.

Sample	Parameters	
	Total Color Difference *dE*	Viscosity (Pa·s)
Control	-	3.77 ± 0.90 ^a^
Flaxseed meal		
F 4%	9.1 ± 0.3 ^c^	14.6 ± 2.0 ^ab^
F 6.5%	7.1 ± 0.8 ^b^	22.4 ± 2.2 ^ab^
F 9%	12.8 ± 0.3 ^d^	40.4 ± 3.0 ^ab^
F 11.5%	14.6 ± 0.4 ^e^	94.4 ± 8.4 ^b^
F 14%	14.9 ± 0.7 ^e^	306 ± 72 ^c^
Chia seed meal		
C 4%	3.8 ± 1.0 ^a^	8.4 ± 4.1 ^ab^
C 6.5%	6.8 ± 0.8 ^b^	20.8 ± 1.5 ^ab^
C 9%	11.7 ± 0.3 ^d^	372 ± 43 ^c^
C 11.5%	7.7 ± 0.8 ^b^	318 ± 19 ^c^
C 14%	7.7 ± 1.0 ^b^	391 ± 55 ^c^

Sample codes: as in Table 2. Mean ± SD. In each column, means marked with different superscript letters are significantly different (one-way ANOVA, Tukey HSD test at α = 0.05).

**Table 5:** Correlation between consumer desirability (consumer analysis) and the intensity of product attribute descriptors determined in sensory profiling analysis. Pearson correlation coefficient r values.

Sensory Profiling		Consumer Desirability	
Attribute	Descriptor	Color	Overall
Color	**green**	0.928	0.931
	**white**	−0.481	−0.514
	**yellow**	−0.606	−0.619
	**grey**	−0.450	0.465
		**taste**	**overall**
Taste	**pickled cucumber**	−0.607	−0.528
	**vegetable**	−0.368	−0.362
	**tart**	−0.769	−0.720
	**sweet**	0.055	0.025
	**bitter**	−0.471	−0.519
	**sour**	−0.755	−0.776
	**metallic**	0.529	0.413
	**strange**	−0.411	−0.687
		**spreadability**	**overall**
Consistency	**uniformity**	0.326	0.326
	**spreadability**	0.893	0.657
	**firmness**	0.527	0.271

## Data Availability

The data presented in this study are included in the article and Supplementary Materials. Further inquiries can be directed to the corresponding authors.

## Supplementary Materials

The following supporting information can be downloaded at: https://www.mdpi.com/article/10.3390/foods14030438/s1, Table S1: Mean scores of consumer desirability attributes (i.e. color, aroma, spreadability, taste, consistency and overall) of the fermented vegetable-based spreads with various chia or flaxseed meal additions and the control (without the additions); Table S2: Mean scores of sensory color and consistency profiling of the fermented vegetable-based spreads with various chia or flaxseed meal additions and the control (without the additions); Table S3: Mean scores of sensory taste profiling of the fermented vegetable-based spreads with various chia or flaxseed additions and the control (without the additions).
