# Supplementary material for: Application of Chia and Flaxseed Meal as an Ingredient of Fermented Vegetable-Based Spreads to Design Their Nutritional Composition and Sensory Quality
Source: Foods. 2025 Jan 29;14(3):438. doi: 10.3390/foods14030438 (PMC11816384; doi:10.3390/foods14030438)
Supplement: Supplementary file 1 [file foods-14-00438-s001.zip › foods-3414808-supplementary.pdf]

Application of Chia and Flaxseed Meal as an Ingredient of Fermented Vegetable-Based Spreads to Design Their Nutritional Composition and Sensory Quality

Katarzyna Waszkowiak [1*], Krystyna Szymandera-Buszka [1], Marcin Kidoń [2], Joanna Kobus-Cisowska[1], Anna Brzozowska [1], Angelika Kowiel [2], Maciej Jarzębski [3], Elżbieta Radziejewska-Kubzdela [2*]

Foods **2025**, *14*, x. https://doi.org/10.3390/xxxxx

Supplementary material

Table S1 Mean scores of consumer desirability attributes (i.e. color, aroma, spreadability, taste, consistency and overall) of the fermented vegetable-based spreads with various chia or flaxseed meal additions and the control (without the additions)

Samples	Desirability					
	COLOR	AROMA	SPREADA-BILITY	TASTE	CONSIST-ENCY	OVERALL
Control	5.40±1.7 [a*]	6.00±1.7 [a]	3.00± [a]	5.50± [a]	4.50±1.7 [a]	5.00±1.8 [a]
Flaxseed meal						
F 4%	5.50±1.8 [a]	6.00±1.6 [a]	3.50±0.8 [a]	5.50±1.4 [a]	4.50±1.6 [a]	5.00±1.7 [a]
F 6.5%	5.60±1.6 [a]	6.20±1.7 [a]	4.00±1.7 [ba]	5.90±1.9 [a]	4.60±1.7 [a]	5.20±1.8 [a]
F 9%	5.60±1.8 [a]	6.00±1.8 [a]	5.00±1.7 [ba]	7.60±1.9 [a]	5.50±1.8 [a]	7.50±1.7 [a]
F 11.5%	7.50±1.6 [b]	6.80±1.5 [a]	6.50±1.6 [b]	7.50±1.0 [a]	6.80±1.7 [a]	7.60±1.8 [a]
F 14%	8.00±1.9 [b]	7.00±1.6 [a]	7.80±0.8 [b]	7.20±1.9 [a]	7.00±1.7 [a]	7.00±1.7 [a]
Chia seed meal						
C 4%	5.50±1.9 [a]	5.90±1.7 [a]	3.20±1.7 [a]	5.60±1.9 [a]	4.60±0.8 [a]	5.10±1.7 [a]
C 6.5%	5.20±1.6 [a]	6.00±0.8 [a]	3.90±1.6 [a]	5.60±1.4 [a]	5.30±1.7 [a]	5.00±1.9 [a]
C 9%	5.00±1.7 [a]	5.50±1.7 [a]	4.50±2.0 [ba]	5.90±1.5 [a]	5.00±1.8 [a]	5.60±2.0 [a]
C 11.5%	5.30±1.8 [a]	6.00±1.6 [a]	4.80±1.5 [ba]	6.00±1.9 [a]	5.00±1.6 [a]	5.00±1.4 [a]
C 14%	5.90±1.6 [a]	5.50±1.0 [a]	4.50±1.4 [ba]	5.80±0.8 [a]	4.50±1.5 [a]	5.20±1.8 [a]

Sample codes: control - the samples without the meal addition; F 4%, F 6.5%, F 9%, F 11.5%, F 14% and C 4%, C 6.5%, C 9%, C 11.5%, C 14% - the samples with 4%, 6.5% 9% 11.5% and 14% addition of flaxseed meal (F) or chia seed meal (C), respectively. * Mean ± SD. In each column, means (n = 62) marked with different superscript letters are significantly different (one-way ANOVA, Tukey HSD test at $\alpha = 0.05$).

Application of Chia and Flaxseed Meal as an Ingredient of Fermented Vegetable-Based Spreads to Design Their Nutritional Composition and Sensory Quality

Katarzyna Waszkowiak [1*], Krystyna Szymandera-Buszka [1], Marcin Kidoń [2], Joanna Kobus-Cisowska[1], Anna Brzozowska [1], Angelika Kowiel [2], Maciej Jarzębski [3], Elżbieta Radziejewska-Kubzdela [2*]

Foods **2025**, *14*, x. https://doi.org/10.3390/xxxxx

Table S2 Mean scores of sensory color and consistency profiling of the fermented vegetable-based spreads with various chia or flaxseed meal additions and the control (without the additions)

Samples	Color descriptors				Consistency descriptors		
	Green	White	Yellow	Grey	Uniformity	Spreadability	Firmness
Control	5.60±0.2 ^{c*}	0.00±0.0 ^{b}	3.50±0.3 ^{cb}	0.50±0.2 ^{b}	2.50±0.3 ^{d}	2.80±0.3 ^{c}	1.00±0.4 ^{d}
Flaxseed meal							
F 4%	5.25±0.4 ^{dc}	0.00±0.0 ^{b}	3.50±0.3 ^{cb}	0.50±0.2 ^{b}	3.20±0.4 ^{dc}	3.00±0.3 ^{c}	1.60±0.4 ^{dc}
F 6.5%	5.50±0.2 ^{c}	0.00±0.0 ^{b}	3.10±0.4 ^{c}	0.75±0.3 ^{b}	4.50±0.3 ^{a}	4.00±0.3 ^{b}	2.00±0.3 ^{c}
F 9%	6.00±0.5 ^{cb}	0.00±0.0 ^{b}	2.50±0.3 ^{c}	0.90±0.2 ^{b}	4.90±0.6 ^{a}	4.20±0.3 ^{b}	2.50±0.5 ^{cb}
F 11.5%	6.95±0.4 ^{ba}	0.00±0.0 ^{b}	2.40±0.3 ^{c}	0.90±0.2 ^{b}	3.50±0.4 ^{cb}	5.30±0.4 ^{a}	2.80±0.3 ^{ba}
F 14%	7.50±0.4 ^{a}	0.00±0.0 ^{b}	1.7±0.2 ^{d}	0.90±0.2 ^{b}	4.00±0.5 ^{ba}	5.40±0.5 ^{a}	3.00±0.5 ^{a}
Chia seed meal							
C 4%	5.50±0.2 ^{c}	5.90±0.5 ^{a}	3.20±0.3 ^{c}	5.60±0.3 ^{a}	3.50±0.3 ^{cb}	3.00±0.5 ^{c}	2.60±.02 ^{b}
C 6.5%	5.20±0.3 ^{c}	6.00±0.3 ^{a}	3.90±0.3 ^{cb}	5.60±0.4 ^{a}	4.20±0.3 ^{ba}	4.20±0.3 ^{b}	3.20±0.3 ^{a}
C 9%	5.00±0.3 ^{d}	5.50±0.4 ^{a}	4.50±0.4 ^{ba}	5.90±0.4 ^{a}	4.00±0.4 ^{ba}	4.70±0.3 ^{ba}	2.80±0.4 ^{ba}
C 11.5%	5.30±0.3 ^{dc}	6.00±0.4 ^{a}	4.80±0.3 ^{a}	6.00±0.4 ^{a}	4.20±0.3 ^{ba}	4.50±0.4 ^{ba}	2.90±0.4 ^{ba}
C 14%	5.00±0.3 ^{d}	5.50±0.2 ^{a}	4.50±0.5 ^{ba}	5.80±0.4 ^{a}	3.70±0.4 ^{b}	4.00±0.4 ^{b}	3.10±0,3 ^{a}

Sample codes: as in Table S1. *Mean ± SD. In each column, means (n = 8) marked with different superscript letters are significantly different (one-way ANOVA, Tukey HSD test at $\alpha = 0.05$).

Table S3 Mean scores of sensory taste profiling of the fermented vegetable-based spreads with various chia or flaxseed additions and the control (without the additions)

Samples	Taste descriptors						
	Pickled	Vegetable	Tart	Sweet	Bitter	Sour	Metallic	Strange
Control	6.80±0.5 ^{a*}	4.20±0.4 ^{b}	4.20±0.3 ^{a}	0.50±0.5 ^{a}	1.00±0.4 ^{c}	3.50±0.4 ^{ba}	0.50±0.3 ^{cb}	0.50±0.2 ^{a}
Flaxseed meal								
F 4%	6.50±0.3 ^{a}	4.00±0.4 ^{b}	4.00±0.4 ^{ba}	0.50±0.3 ^{a}	1.00±0.3 ^{c}	3.50±0.3 ^{b}	0.50±0.4 ^{a}	0.20±0.2 ^{ba}
F 6.5%	5.50±0.4 ^{b}	4.20±0.3 ^{b}	3.50±0.4 ^{cb}	0.60±0.4 ^{a}	0.80±0.3 ^{c}	3.10±0.3 ^{cb}	0.75±0.2 ^{a}	0.20±0.2 ^{ba}
F 9%	5.50±0.3 ^{b}	4.00±0.2 ^{b}	3.50±0.3 ^{cb}	0.75±0.4 ^{a}	0.80±0.4 ^{c}	2.50±0.4 ^{c}	0.70±0.2 ^{a}	0.00±0.0 ^{b}
F 11.5%	4.50±0.4 ^{c}	4.00±0.3 ^{b}	2.80±0.5 ^{dc}	0.50±0.5 ^{a}	0.50±0.2 ^{c}	2.40±0.3 ^{c}	0.70±0.2 ^{a}	0.20±0.3 ^{ba}
F 14%	4.30±0.3 ^{c}	4.20±0.2 ^{b}	2.50±0.2 ^{d}	0.70±0.2 ^{a}	0.50±0.2 ^{c}	1.70±0.2 ^{c}	0.80±0.2 ^{a}	0.20± 0.2 ^{ba}
Chia seed meal								
C 4%	6.20±0.4 ^{ba}	5.00±0.3 ^{a}	4.00±0.4 ^{ba}	0.50±0.2 ^{a}	1.20±0.3 ^{c}	3.20±0.5 ^{b}	0.40±0.3 ^{a}	0.50±0.2 ^{a}
C 6.5%	6.00±0.3 ^{ba}	4.50±0.5 ^{ba}	4.00±0.3 ^{ba}	0.90±04 ^{a}	2.50±0.3 ^{b}	3.90±0.3 ^{ba}	0.30±0.3 ^{a}	0.60±0.3 ^{a}
C 9%	5.40±0.5 ^{cb}	4.00±0.4 ^{b}	3.80±0.3 ^{b}	0.80±0.3 ^{a}	3.00±0.3 ^{a}	4.00±0.3 ^{a}	0.50±0.3 ^{a}	0.50±0.3 ^{a}
C 11.5%	4.80±0.5 ^{c}	4.20±0.3 ^{b}	3.50±0.4 ^{cb}	0.80±0.5 ^{a}	3.00±0.3 ^{a}	4.20±0.4 ^{a}	0.80±0.4 ^{a}	0.50±0.2 ^{a}
C 14%	4.50±0.4 ^{c}	4.00±0.2 ^{b}	3.30±0.3 ^{c}	0.70±0.2 ^{a}	3.20±0.3 ^{a}	3.00±0.4 ^{cb}	0.80±0.3 ^{a}	0.70±0.3 ^{a}

Sample codes: as in Table S1. *Mean ± SD. In each column, means (n = 8) marked with different superscript letters are significantly different (one-way ANOVA, Tukey HSD test at $\alpha = 0.05$).
